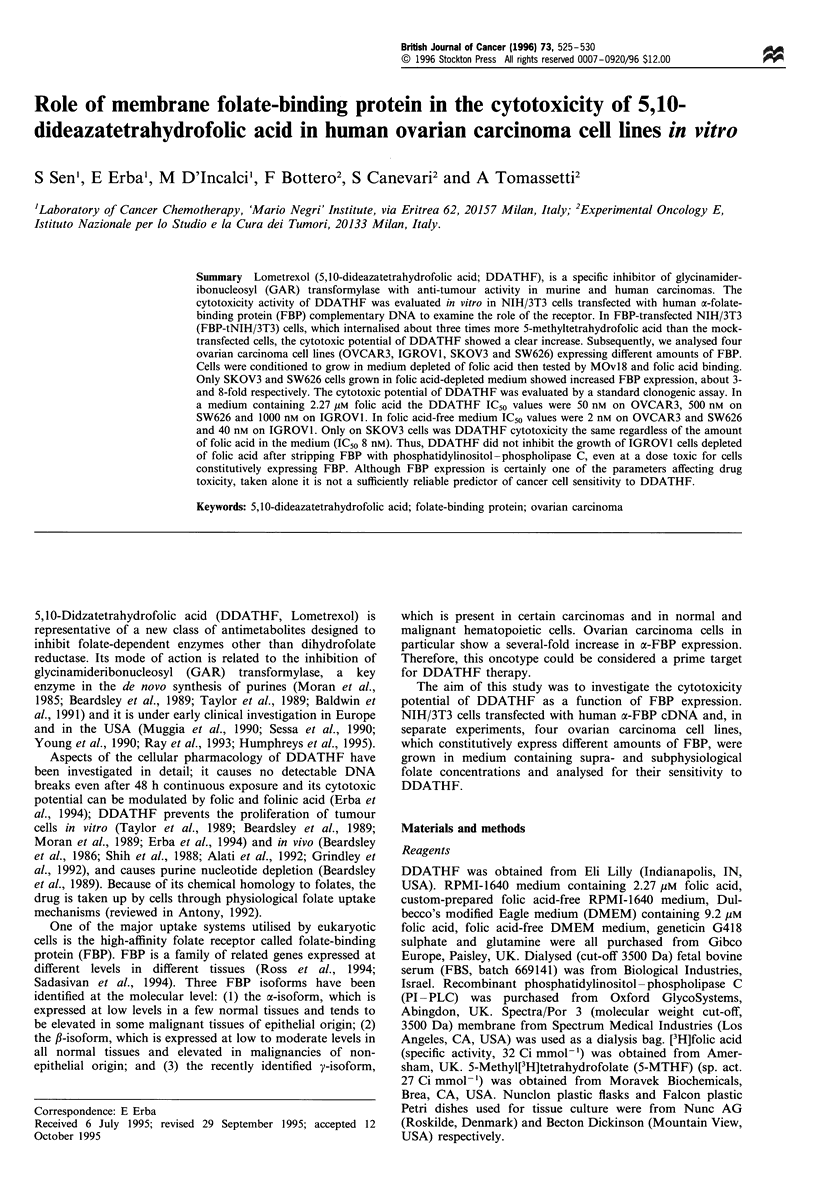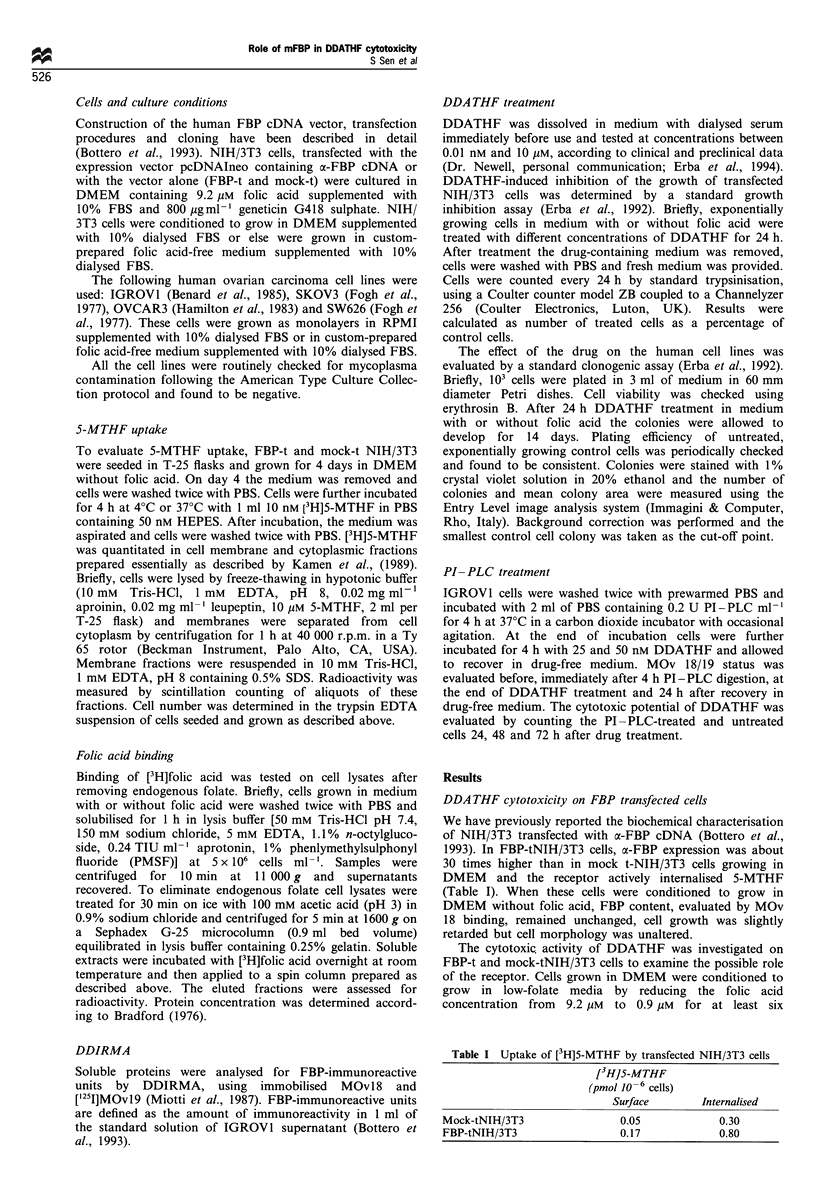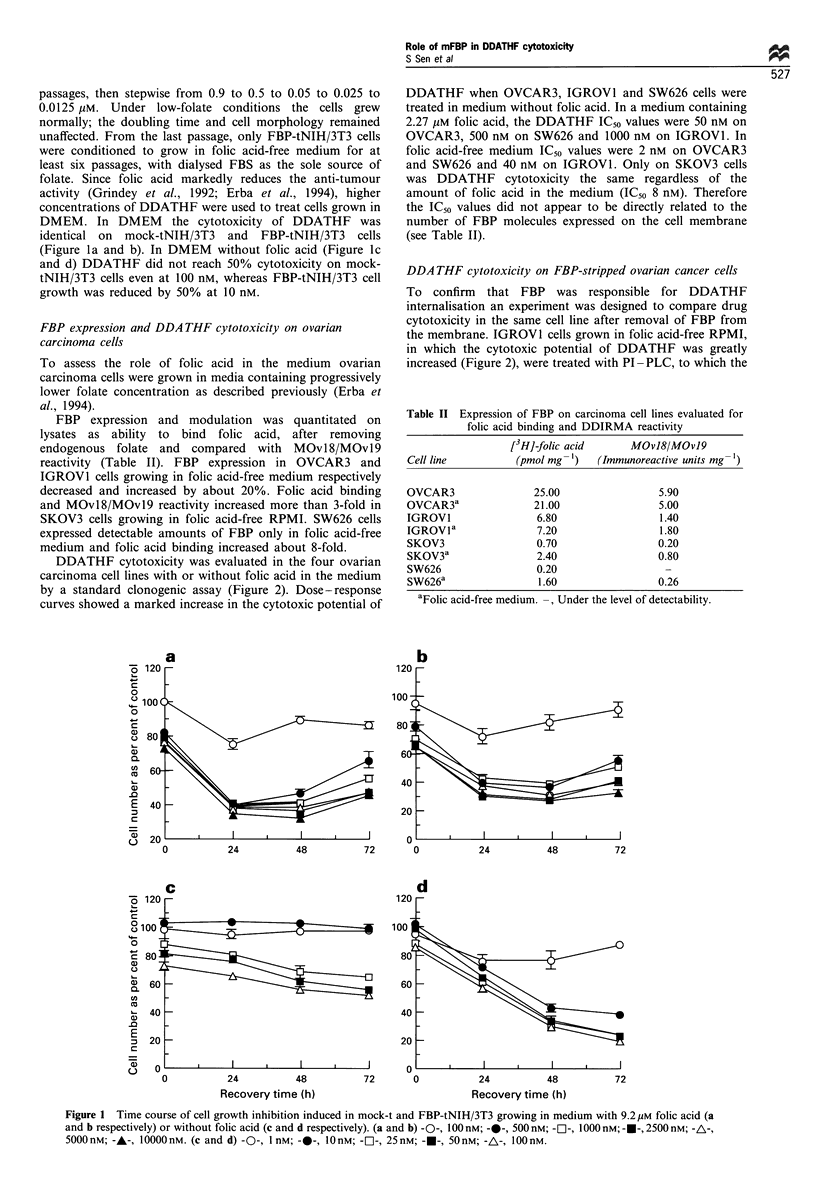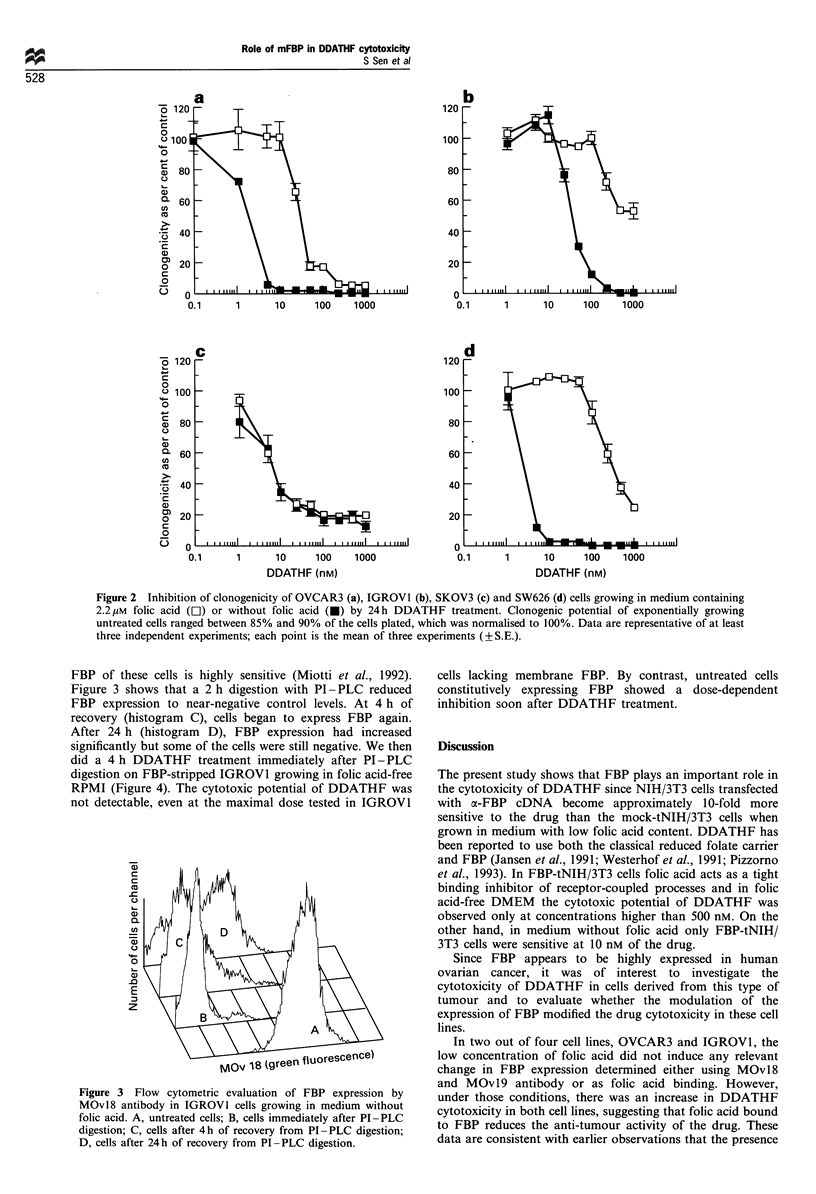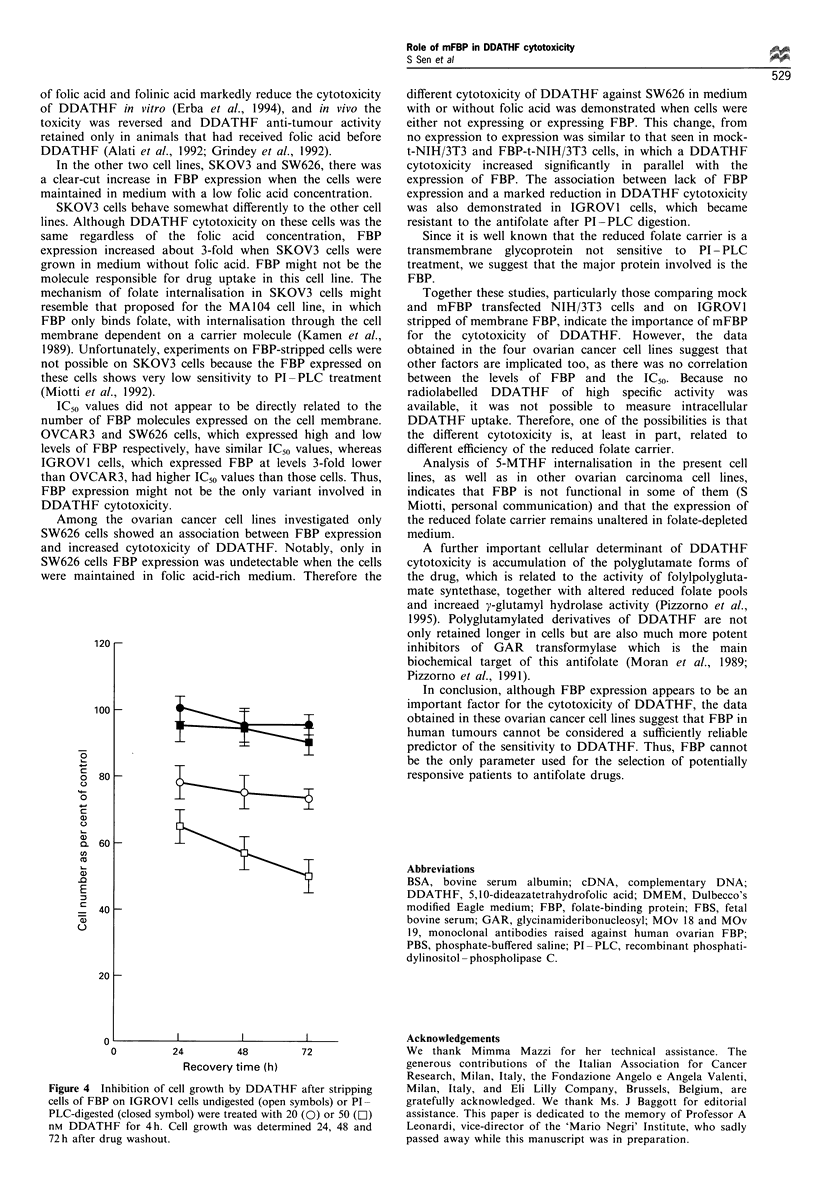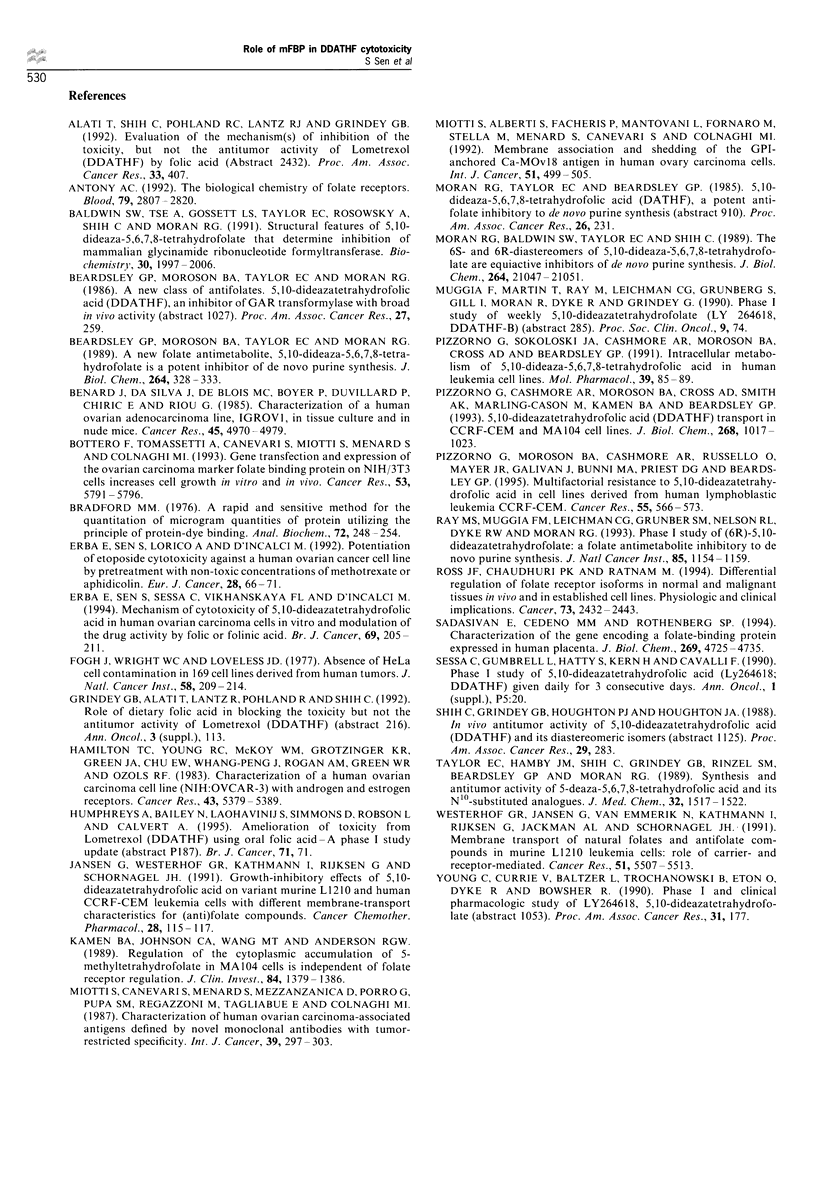# Role of membrane folate-binding protein in the cytotoxicity of 5,10-dideazatetrahydrofolic acid in human ovarian carcinoma cell lines in vitro.

**DOI:** 10.1038/bjc.1996.91

**Published:** 1996-02

**Authors:** S. Sen, E. Erba, M. D'Incalci, F. Bottero, S. Canevari, A. Tomassetti

**Affiliations:** Laboratory of Cancer Chemotherapy, 'Mario Negri' Institute, Milan, Italy.

## Abstract

Lometrexol (5,10-dideazatetrahydrofolic acid; DDATHF), is a specific inhibitor of glycinamideribonucleosyl (GAR) transformylase with anti-tumour activity in murine and human carcinomas. The cytotoxicity activity of DDATHF was evaluated in vitro in NIH/3T3 cells transfected with human alpha-folate-binding protein (FBP) complementary DNA to examine the role of the receptor. In FBP-transfected NIH/3T3 (FBP-tNIH/3T3) cells, which internalised about three times more 5-methyltetrahydrofolic acid than the mock-transfected cells, the cytotoxtic potential of DDATHF showed a clear increase. Subsequently, we analysed four ovarian carcinoma cell lines (OVCAR3, IGROV1, SKOV3, and SW626) expressing different amounts of FBP. Cells were conditioned to grow in medium depleted of folic acid then tested by MOv18 and folic acid binding. Only SKOV3 and SW626 cells grown in folic acid-depleted medium showed increased FBP expression, about 3- and 8-fold respectively. The cytotoxic potential of DDATHF was evaluated by a standard clonogenic assay. In a medium containing 2.27 microM folic acid the DDATHF IC50 values were 50 nm on OVCAR3, 500 nM on SW626 and 1000 nM on IGROV1. In folic acid-free medium IC50 values were 2 nM on OVCAR3 and Sw626 and 40 nM on IGROV1. Only on SKOV3 cells was DDATHF cytotoxicity the same regardless of the amount of folic acid in the medium (IC50 8 nM). Thus, DDATHF did not inhibit the growth of IGROV1 cells depleted of folic acid after stripping FBP with phosphatidylinositol-phospholipase C, even at a dose toxic for cells constitutively expressing FBP. Although FBP expression is certainly one of the parameters affecting drug toxicity, taken alone it is not a sufficiently reliable predictor of cancer cell sensitivity to DDATHF.


British Journal of Cancer (1996) 73, 525-530
© 1996 Stockton Press All rights reserved 0007-0920/96 $12.00

### Role of membrane folate-binding protein in the cytotoxicity of 5,10-dideazatetrahydrofolic acid in human ovarian carcinoma cell lines *in vitro*

S Sen[1], E Erba[1], M D'Incalci[1], F Bottero[2], S Canevari[2] and A Tomassetti[2]

[1]*Laboratory of Cancer Chemotherapy, 'Mario Negri' Institute, via Eritrea 62, 20157 Milan, Italy;* [2]*Experimental Oncology E, Istituto Nazionale per lo Studio e la Cura dei Tumori, 20133 Milan, Italy.*

**Summary** Lometrexol (5,10-dideazatetrahydrofolic acid; DDATHF), is a specific inhibitor of glycinamideribonucleosyl (GAR) transformylase with anti-tumour activity in murine and human carcinomas. The cytotoxicity activity of DDATHF was evaluated *in vitro* in NIH/3T3 cells transfected with human α-folate-binding protein (FBP) complementary DNA to examine the role of the receptor. In FBP-transfected NIH/3T3 (FBP-tNIH/3T3) cells, which internalised about three times more 5-methyltetrahydrofolic acid than the mock-transfected cells, the cytotoxic potential of DDATHF showed a clear increase. Subsequently, we analysed four ovarian carcinoma cell lines (OVCAR3, IGROV1, SKOV3 and SW626) expressing different amounts of FBP. Cells were conditioned to grow in medium depleted of folic acid then tested by MOv18 and folic acid binding. Only SKOV3 and SW626 cells grown in folic acid-depleted medium showed increased FBP expression, about 3- and 8-fold respectively. The cytotoxic potential of DDATHF was evaluated by a standard clonogenic assay. In a medium containing 2.27 μM folic acid the DDATHF $IC_{50}$ values were 50 nM on OVCAR3, 500 nM on SW626 and 1000 nM on IGROV1. In folic acid-free medium $IC_{50}$ values were 2 nM on OVCAR3 and SW626 and 40 nM on IGROV1. Only on SKOV3 cells was DDATHF cytotoxicity the same regardless of the amount of folic acid in the medium ($IC_{50}$ 8 nM). Thus, DDATHF did not inhibit the growth of IGROV1 cells depleted of folic acid after stripping FBP with phosphatidylinositol–phospholipase C, even at a dose toxic for cells constitutively expressing FBP. Although FBP expression is certainly one of the parameters affecting drug toxicity, taken alone it is not a sufficiently reliable predictor of cancer cell sensitivity to DDATHF.

**Keywords:** 5,10-dideazatetrahydrofolic acid; folate-binding protein; ovarian carcinoma

5,10-Didzatetrahydrofolic acid (DDATHF, Lometrexol) is representative of a new class of antimetabolites designed to inhibit folate-dependent enzymes other than dihydrofolate reductase. Its mode of action is related to the inhibition of glycinamideribonucleosyl (GAR) transformylase, a key enzyme in the *de novo* synthesis of purines (Moran *et al.*, 1985; Beardsley *et al.*, 1989; Taylor *et al.*, 1989; Baldwin *et al.*, 1991) and it is under early clinical investigation in Europe and in the USA (Muggia *et al.*, 1990; Sessa *et al.*, 1990; Young *et al.*, 1990; Ray *et al.*, 1993; Humphreys *et al.*, 1995).

Aspects of the cellular pharmacology of DDATHF have been investigated in detail; it causes no detectable DNA breaks even after 48 h continuous exposure and its cytotoxic potential can be modulated by folic and folinic acid (Erba *et al.*, 1994); DDATHF prevents the proliferation of tumour cells *in vitro* (Taylor *et al.*, 1989; Beardsley *et al.*, 1989; Moran *et al.*, 1989; Erba *et al.*, 1994) and *in vivo* (Beardsley *et al.*, 1986; Shih *et al.*, 1988; Alati *et al.*, 1992; Grindley *et al.*, 1992), and causes purine nucleotide depletion (Beardsley *et al.*, 1989). Because of its chemical homology to folates, the drug is taken up by cells through physiological folate uptake mechanisms (reviewed in Antony, 1992).

One of the major uptake systems utilised by eukaryotic cells is the high-affinity folate receptor called folate-binding protein (FBP). FBP is a family of related genes expressed at different levels in different tissues (Ross *et al.*, 1994; Sadasivan *et al.*, 1994). Three FBP isoforms have been identified at the molecular level: (1) the α-isoform, which is expressed at low levels in a few normal tissues and tends to be elevated in some malignant tissues of epithelial origin; (2) the β-isoform, which is expressed at low to moderate levels in all normal tissues and elevated in malignancies of non-epithelial origin; and (3) the recently identified γ-isoform, which is present in certain carcinomas and in normal and malignant hematopoietic cells. Ovarian carcinoma cells in particular show a several-fold increase in α-FBP expression. Therefore, this oncotype could be considered a prime target for DDATHF therapy.

The aim of this study was to investigate the cytotoxicity potential of DDATHF as a function of FBP expression. NIH/3T3 cells transfected with human α-FBP cDNA and, in separate experiments, four ovarian carcinoma cell lines, which constitutively express different amounts of FBP, were grown in medium containing supra- and subphysiological folate concentrations and analysed for their sensitivity to DDATHF.

### Materials and methods

### *Reagents*

DDATHF was obtained from Eli Lilly (Indianapolis, IN, USA). RPMI-1640 medium containing 2.27 μM folic acid, custom-prepared folic acid-free RPMI-1640 medium, Dulbecco's modified Eagle medium (DMEM) containing 9.2 μM folic acid, folic acid-free DMEM medium, geneticin G418 sulphate and glutamine were all purchased from Gibco Europe, Paisley, UK. Dialysed (cut-off 3500 Da) fetal bovine serum (FBS, batch 669141) was from Biological Industries, Israel. Recombinant phosphatidylinositol–phospholipase C (PI–PLC) was purchased from Oxford GlycoSystems, Abingdon, UK. Spectra/Por 3 (molecular weight cut-off, 3500 Da) membrane from Spectrum Medical Industries (Los Angeles, CA, USA) was used as a dialysis bag. [$^3$H]folic acid (specific activity, 32 Ci $mmol^{-1}$) was obtained from Amersham, UK. 5-Methyl[$^3$H]tetrahydrofolate (5-MTHF) (sp. act. 27 Ci $mmol^{-1}$) was obtained from Moravek Biochemicals, Brea, CA, USA. Nunclon plastic flasks and Falcon plastic Petri dishes used for tissue culture were from Nunc AG (Roskilde, Denmark) and Becton Dickinson (Mountain View, USA) respectively.

Correspondence: E Erba
Received 6 July 1995; revised 29 September 1995; accepted 12 October 1995

*Cells and culture conditions*

Construction of the human FBP cDNA vector, transfection procedures and cloning have been described in detail (Bottero *et al.*, 1993). NIH/3T3 cells, transfected with the expression vector pcDNAIneo containing α-FBP cDNA or with the vector alone (FBP-t and mock-t) were cultured in DMEM containing 9.2 μM folic acid supplemented with 10% FBS and 800 $\mu g ml^{-1}$ geneticin G418 sulphate. NIH/3T3 cells were conditioned to grow in DMEM supplemented with 10% dialysed FBS or else were grown in custom-prepared folic acid-free medium supplemented with 10% dialysed FBS.

The following human ovarian carcinoma cell lines were used: IGROV1 (Benard *et al.*, 1985), SKOV3 (Fogh *et al.*, 1977), OVCAR3 (Hamilton *et al.*, 1983) and SW626 (Fogh *et al.*, 1977). These cells were grown as monolayers in RPMI supplemented with 10% dialysed FBS or in custom-prepared folic acid-free medium supplemented with 10% dialysed FBS.

All the cell lines were routinely checked for mycoplasma contamination following the American Type Culture Collection protocol and found to be negative.

*5-MTHF uptake*

To evaluate 5-MTHF uptake, FBP-t and mock-t NIH/3T3 were seeded in T-25 flasks and grown for 4 days in DMEM without folic acid. On day 4 the medium was removed and cells were washed twice with PBS. Cells were further incubated for 4 h at 4°C or 37°C with 1 ml 10 nM [$^3$H]5-MTHF in PBS containing 50 nM HEPES. After incubation, the medium was aspirated and cells were washed twice with PBS. [$^3$H]5-MTHF was quantitated in cell membrane and cytoplasmic fractions prepared essentially as described by Kamen *et al.*, (1989). Briefly, cells were lysed by freeze-thawing in hypotonic buffer (10 mM Tris-HCl, 1 mM EDTA, pH 8, 0.02 mg $ml^{-1}$ aproinin, 0.02 mg $ml^{-1}$ leupeptin, 10 μM 5-MTHF, 2 ml per T-25 flask) and membranes were separated from cell cytoplasm by centrifugation for 1 h at 40 000 r.p.m. in a Ty 65 rotor (Beckman Instrument, Palo Alto, CA, USA). Membrane fractions were resuspended in 10 mM Tris-HCl, 1 mM EDTA, pH 8 containing 0.5% SDS. Radioactivity was measured by scintillation counting of aliquots of these fractions. Cell number was determined in the trypsin EDTA suspension of cells seeded and grown as described above.

*Folic acid binding*

Binding of [$^3$H]folic acid was tested on cell lysates after removing endogenous folate. Briefly, cells grown in medium with or without folic acid were washed twice with PBS and solubilised for 1 h in lysis buffer [50 mM Tris-HCl pH 7.4, 150 mM sodium chloride, 5 mM EDTA, 1.1% *n*-octylglucoside, 0.24 TIU $ml^{-1}$ aprotonin, 1% phenlymethylsulphonyl fluoride (PMSF)] at $5 \times 10^6$ cells $ml^{-1}$. Samples were centrifuged for 10 min at 11 000 *g* and supernatants recovered. To eliminate endogenous folate cell lysates were treated for 30 min on ice with 100 mM acetic acid (pH 3) in 0.9% sodium chloride and centrifuged for 5 min at 1600 *g* on a Sephadex G-25 microcolumn (0.9 ml bed volume) equilibrated in lysis buffer containing 0.25% gelatin. Soluble extracts were incubated with [$^3$H]folic acid overnight at room temperature and then applied to a spin column prepared as described above. The eluted fractions were assessed for radioactivity. Protein concentration was determined according to Bradford (1976).

*DDIRMA*

Soluble proteins were analysed for FBP-immunoreactive units by DDIRMA, using immobilised MOv18 and [$^{125}$I]MOv19 (Miotti *et al.*, 1987). FBP-immunoreactive units are defined as the amount of immunoreactivity in 1 ml of the standard solution of IGROV1 supernatant (Bottero *et al.*, 1993).

*DDATHF treatment*

DDATHF was dissolved in medium with dialysed serum immediately before use and tested at concentrations between 0.01 nM and 10 μM, according to clinical and preclinical data (Dr. Newell, personal communication; Erba *et al.*, 1994). DDATHF-induced inhibition of the growth of transfected NIH/3T3 cells was determined by a standard growth inhibition assay (Erba *et al.*, 1992). Briefly, exponentially growing cells in medium with or without folic acid were treated with different concentrations of DDATHF for 24 h. After treatment the drug-containing medium was removed, cells were washed with PBS and fresh medium was provided. Cells were counted every 24 h by standard trypsinisation, using a Coulter counter model ZB coupled to a Channelyzer 256 (Coulter Electronics, Luton, UK). Results were calculated as number of treated cells as a percentage of control cells.

The effect of the drug on the human cell lines was evaluated by a standard clonogenic assay (Erba *et al.*, 1992). Briefly, $10^3$ cells were plated in 3 ml of medium in 60 mm diameter Petri dishes. Cell viability was checked using erythrosin B. After 24 h DDATHF treatment in medium with or without folic acid the colonies were allowed to develop for 14 days. Plating efficiency of untreated, exponentially growing control cells was periodically checked and found to be consistent. Colonies were stained with 1% crystal violet solution in 20% ethanol and the number of colonies and mean colony area were measured using the Entry Level image analysis system (Immagini & Computer, Rho, Italy). Background correction was performed and the smallest control cell colony was taken as the cut-off point.

*PI–PLC treatment*

IGROV1 cells were washed twice with prewarmed PBS and incubated with 2 ml of PBS containing 0.2 U PI–PLC $ml^{-1}$ for 4 h at 37°C in a carbon dioxide incubator with occasional agitation. At the end of incubation cells were further incubated for 4 h with 25 and 50 nM DDATHF and allowed to recover in drug-free medium. MOv 18/19 status was evaluated before, immediately after 4 h PI–PLC digestion, at the end of DDATHF treatment and 24 h after recovery in drug-free medium. The cytotoxic potential of DDATHF was evaluated by counting the PI–PLC-treated and untreated cells 24, 48 and 72 h after drug treatment.

### Results

*DDATHF cytotoxicity on FBP transfected cells*

We have previously reported the biochemical characterisation of NIH/3T3 transfected with α-FBP cDNA (Bottero *et al.*, 1993). In FBP-tNIH/3T3 cells, α-FBP expression was about 30 times higher than in mock t-NIH/3T3 cells growing in DMEM and the receptor actively internalised 5-MTHF (Table I). When these cells were conditioned to grow in DMEM without folic acid, FBP content, evaluated by MOv 18 binding, remained unchanged, cell growth was slightly retarded but cell morphology was unaltered.

The cytotoxic activity of DDATHF was investigated on FBP-t and mock-tNIH/3T3 cells to examine the possible role of the receptor. Cells grown in DMEM were conditioned to grow in low-folate media by reducing the folic acid concentration from 9.2 μM to 0.9 μM for at least six

**Table I** Uptake of [$^3$H]5-MTHF by transfected NIH/3T3 cells

| | [$^3$H]5-MTHF (pmol $10^{-6}$ cells) Surface | Internalised |
|---|---|---|
| Mock-tNIH/3T3 | 0.05 | 0.30 |
| FBP-tNIH/3T3 | 0.17 | 0.80 |

passages, then stepwise from 0.9 to 0.5 to 0.05 to 0.025 to 0.0125 μM. Under low-folate conditions the cells grew normally; the doubling time and cell morphology remained unaffected. From the last passage, only FBP-tNIH/3T3 cells were conditioned to grow in folic acid-free medium for at least six passages, with dialysed FBS as the sole source of folate. Since folic acid markedly reduces the anti-tumour activity (Grindey *et al.*, 1992; Erba *et al.*, 1994), higher concentrations of DDATHF were used to treat cells grown in DMEM. In DMEM the cytotoxicity of DDATHF was identical on mock-tNIH/3T3 and FBP-tNIH/3T3 cells (Figure 1a and b). In DMEM without folic acid (Figure 1c and d) DDATHF did not reach 50% cytotoxicity on mock-tNIH/3T3 cells even at 100 nM, whereas FBP-tNIH/3T3 cell growth was reduced by 50% at 10 nM.

### *FBP expression and DDATHF cytotoxicity on ovarian carcinoma cells*

To assess the role of folic acid in the medium ovarian carcinoma cells were grown in media containing progressively lower folate concentration as described previously (Erba *et al.*, 1994).

FBP expression and modulation was quantitated on lysates as ability to bind folic acid, after removing endogenous folate and compared with MOv18/MOv19 reactivity (Table II). FBP expression in OVCAR3 and IGROV1 cells growing in folic acid-free medium respectively decreased and increased by about 20%. Folic acid binding and MOv18/MOv19 reactivity increased more than 3-fold in SKOV3 cells growing in folic acid-free RPMI. SW626 cells expressed detectable amounts of FBP only in folic acid-free medium and folic acid binding increased about 8-fold.

DDATHF cytotoxicity was evaluated in the four ovarian carcinoma cell lines with or without folic acid in the medium by a standard clonogenic assay (Figure 2). Dose–response curves showed a marked increase in the cytotoxic potential of DDATHF when OVCAR3, IGROV1 and SW626 cells were treated in medium without folic acid. In a medium containing 2.27 μM folic acid, the DDATHF $IC_{50}$ values were 50 nM on OVCAR3, 500 nM on SW626 and 1000 nM on IGROV1. In folic acid-free medium $IC_{50}$ values were 2 nM on OVCAR3 and SW626 and 40 nM on IGROV1. Only on SKOV3 cells was DDATHF cytotoxicity the same regardless of the amount of folic acid in the medium ($IC_{50}$ 8 nM). Therefore the $IC_{50}$ values did not appear to be directly related to the number of FBP molecules expressed on the cell membrane (see Table II).

### *DDATHF cytotoxicity on FBP-stripped ovarian cancer cells*

To confirm that FBP was responsible for DDATHF internalisation an experiment was designed to compare drug cytotoxicity in the same cell line after removal of FBP from the membrane. IGROV1 cells grown in folic acid-free RPMI, in which the cytotoxic potential of DDATHF was greatly increased (Figure 2), were treated with PI–PLC, to which the

**Table II** Expression of FBP on carcinoma cell lines evaluated for folic acid binding and DDIRMA reactivity

| *Cell line* | *[$^3$H]-folic acid (pmol mg$^{-1}$)* | *MOv18/MOv19 (Immunoreactive units mg$^{-1}$)* |
|---|---|---|
| OVCAR3 | 25.00 | 5.90 |
| OVCAR3[a] | 21.00 | 5.00 |
| IGROV1 | 6.80 | 1.40 |
| IGROV1[a] | 7.20 | 1.80 |
| SKOV3 | 0.70 | 0.20 |
| SKOV3[a] | 2.40 | 0.80 |
| SW626 | 0.20 | – |
| SW626[a] | 1.60 | 0.26 |

[a]Folic acid-free medium. –, Under the level of detectability.

**Figure 1** Time course of cell growth inhibition induced in mock-t and FBP-tNIH/3T3 growing in medium with 9.2 μM folic acid (**a** and **b** respectively) or without folic acid (**c** and **d** respectively). (**a** and **b**) -○-, 100 nM; -●-, 500 nM; -□-, 1000 nM; -■-, 2500 nM; -△-, 5000 nM; -▲-, 10000 nM. (**c** and **d**) -○-, 1 nM; -●-, 10 nM; -□-, 25 nM; -■-, 50 nM; -△-, 100 nM.

**Figure 2** Inhibition of clonogenicity of OVCAR3 (**a**), IGROV1 (**b**), SKOV3 (**c**) and SW626 (**d**) cells growing in medium containing 2.2 $\mu$M folic acid (□) or without folic acid (■) by 24 h DDATHF treatment. Clonogenic potential of exponentially growing untreated cells ranged between 85% and 90% of the cells plated, which was normalised to 100%. Data are representative of at least three independent experiments; each point is the mean of three experiments (±S.E.).

FBP of these cells is highly sensitive (Miotti *et al.*, 1992). Figure 3 shows that a 2 h digestion with PI–PLC reduced FBP expression to near-negative control levels. At 4 h of recovery (histogram C), cells began to express FBP again. After 24 h (histogram D), FBP expression had increased significantly but some of the cells were still negative. We then did a 4 h DDATHF treatment immediately after PI–PLC digestion on FBP-stripped IGROV1 growing in folic acid-free RPMI (Figure 4). The cytotoxic potential of DDATHF was not detectable, even at the maximal dose tested in IGROV1 cells lacking membrane FBP. By contrast, untreated cells constitutively expressing FBP showed a dose-dependent inhibition soon after DDATHF treatment.

**Figure 3** Flow cytometric evaluation of FBP expression by MOv18 antibody in IGROV1 cells growing in medium without folic acid. A, untreated cells; B, cells immediately after PI–PLC digestion; C, cells after 4 h of recovery from PI–PLC digestion; D, cells after 24 h of recovery from PI–PLC digestion.

### Discussion

The present study shows that FBP plays an important role in the cytotoxicity of DDATHF since NIH/3T3 cells transfected with α-FBP cDNA become approximately 10-fold more sensitive to the drug than the mock-tNIH/3T3 cells when grown in medium with low folic acid content. DDATHF has been reported to use both the classical reduced folate carrier and FBP (Jansen *et al.*, 1991; Westerhof *et al.*, 1991; Pizzorno *et al.*, 1993). In FBP-tNIH/3T3 cells folic acid acts as a tight binding inhibitor of receptor-coupled processes and in folic acid-free DMEM the cytotoxic potential of DDATHF was observed only at concentrations higher than 500 nM. On the other hand, in medium without folic acid only FBP-tNIH/3T3 cells were sensitive at 10 nM of the drug.

Since FBP appears to be highly expressed in human ovarian cancer, it was of interest to investigate the cytotoxicity of DDATHF in cells derived from this type of tumour and to evaluate whether the modulation of the expression of FBP modified the drug cytotoxicity in these cell lines.

In two out of four cell lines, OVCAR3 and IGROV1, the low concentration of folic acid did not induce any relevant change in FBP expression determined either using MOv18 and MOv19 antibody or as folic acid binding. However, under those conditions, there was an increase in DDATHF cytotoxicity in both cell lines, suggesting that folic acid bound to FBP reduces the anti-tumour activity of the drug. These data are consistent with earlier observations that the presence of folic acid and folinic acid markedly reduce the cytotoxicity of DDATHF *in vitro* (Erba *et al.*, 1994), and *in vivo* the toxicity was reversed and DDATHF anti-tumour activity retained only in animals that had received folic acid before DDATHF (Alati *et al.*, 1992; Grindey *et al.*, 1992).

In the other two cell lines, SKOV3 and SW626, there was a clear-cut increase in FBP expression when the cells were maintained in medium with a low folic acid concentration.

SKOV3 cells behave somewhat differently to the other cell lines. Although DDATHF cytotoxicity on these cells was the same regardless of the folic acid concentration, FBP expression increased about 3-fold when SKOV3 cells were grown in medium without folic acid. FBP might not be the molecule responsible for drug uptake in this cell line. The mechanism of folate internalisation in SKOV3 cells might resemble that proposed for the MA104 cell line, in which FBP only binds folate, with internalisation through the cell membrane dependent on a carrier molecule (Kamen *et al.*, 1989). Unfortunately, experiments on FBP-stripped cells were not possible on SKOV3 cells because the FBP expressed on these cells shows very low sensitivity to PI–PLC treatment (Miotti *et al.*, 1992).

$IC_{50}$ values did not appear to be directly related to the number of FBP molecules expressed on the cell membrane. OVCAR3 and SW626 cells, which expressed high and low levels of FBP respectively, have similar $IC_{50}$ values, whereas IGROV1 cells, which expressed FBP at levels 3-fold lower than OVCAR3, had higher $IC_{50}$ values than those cells. Thus, FBP expression might not be the only variant involved in DDATHF cytotoxicity.

Among the ovarian cancer cell lines investigated only SW626 cells showed an association between FBP expression and increased cytotoxicity of DDATHF. Notably, only in SW626 cells FBP expression was undetectable when the cells were maintained in folic acid-rich medium. Therefore the different cytotoxicity of DDATHF against SW626 in medium with or without folic acid was demonstrated when cells were either not expressing or expressing FBP. This change, from no expression to expression was similar to that seen in mock-t-NIH/3T3 and FBP-t-NIH/3T3 cells, in which a DDATHF cytotoxicity increased significantly in parallel with the expression of FBP. The association between lack of FBP expression and a marked reduction in DDATHF cytotoxicity was also demonstrated in IGROV1 cells, which became resistant to the antifolate after PI–PLC digestion.

Since it is well known that the reduced folate carrier is a transmembrane glycoprotein not sensitive to PI–PLC treatment, we suggest that the major protein involved is the FBP.

Together these studies, particularly those comparing mock and mFBP transfected NIH/3T3 cells and on IGROV1 stripped of membrane FBP, indicate the importance of mFBP for the cytotoxicity of DDATHF. However, the data obtained in the four ovarian cancer cell lines suggest that other factors are implicated too, as there was no correlation between the levels of FBP and the $IC_{50}$. Because no radiolabelled DDATHF of high specific activity was available, it was not possible to measure intracellular DDATHF uptake. Therefore, one of the possibilities is that the different cytotoxicity is, at least in part, related to different efficiency of the reduced folate carrier.

Analysis of 5-MTHF internalisation in the present cell lines, as well as in other ovarian carcinoma cell lines, indicates that FBP is not functional in some of them (S Miotti, personal communication) and that the expression of the reduced folate carrier remains unaltered in folate-depleted medium.

A further important cellular determinant of DDATHF cytotoxicity is accumulation of the polyglutamate forms of the drug, which is related to the activity of folylpolyglutamate syntethase, together with altered reduced folate pools and increaed $\gamma$-glutamyl hydrolase activity (Pizzorno *et al.*, 1995). Polyglutamylated derivatives of DDATHF are not only retained longer in cells but are also much more potent inhibitors of GAR transformylase which is the main biochemical target of this antifolate (Moran *et al.*, 1989; Pizzorno *et al.*, 1991).

In conclusion, although FBP expression appears to be an important factor for the cytotoxicity of DDATHF, the data obtained in these ovarian cancer cell lines suggest that FBP in human tumours cannot be considered a sufficiently reliable predictor of the sensitivity to DDATHF. Thus, FBP cannot be the only parameter used for the selection of potentially responsive patients to antifolate drugs.

**Figure 4** Inhibition of cell growth by DDATHF after stripping cells of FBP on IGROV1 cells undigested (open symbols) or PI–PLC-digested (closed symbol) were treated with 20 (○) or 50 (□) nM DDATHF for 4 h. Cell growth was determined 24, 48 and 72 h after drug washout.

**Abbreviations**

BSA, bovine serum albumin; cDNA, complementary DNA; DDATHF, 5,10-dideazatetrahydrofolic acid; DMEM, Dulbecco's modified Eagle medium; FBP, folate-binding protein; FBS, fetal bovine serum; GAR, glycinamideribonucleosyl; MOv 18 and MOv 19, monoclonal antibodies raised against human ovarian FBP; PBS, phosphate-buffered saline; PI–PLC, recombinant phosphatidylinositol–phospholipase C.

**Acknowledgements**

We thank Mimma Mazzi for her technical assistance. The generous contributions of the Italian Association for Cancer Research, Milan, Italy, the Fondazione Angelo e Angela Valenti, Milan, Italy, and Eli Lilly Company, Brussels, Belgium, are gratefully acknowledged. We thank Ms. J Baggott for editorial assistance. This paper is dedicated to the memory of Professor A Leonardi, vice-director of the 'Mario Negri' Institute, who sadly passed away while this manuscript was in preparation.

### References

ALATI T, SHIH C, POHLAND RC, LANTZ RJ AND GRINDEY GB. (1992). Evaluation of the mechanism(s) of inhibition of the toxicity, but not the antitumor activity of Lometrexol (DDATHF) by folic acid (Abstract 2432). *Proc. Am. Assoc. Cancer Res.*, **33,** 407.

ANTONY AC. (1992). The biological chemistry of folate receptors. *Blood*, **79,** 2807 – 2820.

BALDWIN SW, TSE A, GOSSETT LS, TAYLOR EC, ROSOWSKY A, SHIH C AND MORAN RG. (1991). Structural features of 5,10-dideaza-5,6,7,8-tetrahydrofolate that determine inhibition of mammalian glycinamide ribonucleotide formyltransferase. *Biochemistry*, **30,** 1997 – 2006.

BEARDSLEY GP, MOROSON BA, TAYLOR EC AND MORAN RG. (1986). A new class of antifolates. 5,10-dideazatetrahydrofolic acid (DDATHF), an inhibitor of GAR transformylase with broad *in vivo* activity (abstract 1027). *Proc. Am. Assoc. Cancer Res.*, **27,** 259.

BEARDSLEY GP, MOROSON BA, TAYLOR EC AND MORAN RG. (1989). A new folate antimetabolite, 5,10-dideaza-5,6,7,8-tetrahydrofolate is a potent inhibitor of de novo purine synthesis. *J. Biol. Chem.*, **264,** 328 – 333.

BENARD J, DA SILVA J, DE BLOIS MC, BOYER P, DUVILLARD P, CHIRIC E AND RIOU G. (1985). Characterization of a human ovarian adenocarcinoma line, IGROV1, in tissue culture and in nude mice. *Cancer Res.*, **45,** 4970 – 4979.

BOTTERO F, TOMASSETTI A, CANEVARI S, MIOTTI S, MENARD S AND COLNAGHI MI. (1993). Gene transfection and expression of the ovarian carcinoma marker folate binding protein on NIH/3T3 cells increases cell growth *in vitro* and *in vivo*. *Cancer Res.*, **53,** 5791 – 5796.

BRADFORD MM. (1976). A rapid and sensitive method for the quantitation of microgram quantities of protein utilizing the principle of protein-dye binding. *Anal. Biochem.*, **72,** 248 – 254.

ERBA E, SEN S, LORICO A AND D'INCALCI M. (1992). Potentiation of etoposide cytotoxicity against a human ovarian cancer cell line by pretreatment with non-toxic concentrations of methotrexate or aphidicolin. *Eur. J. Cancer*, **28,** 66 – 71.

ERBA E, SEN S, SESSA C, VIKHANSKAYA FL AND D'INCALCI M. (1994). Mechanism of cytotoxicity of 5,10-dideazatetrahydrofolic acid in human ovarian carcinoma cells in vitro and modulation of the drug activity by folic or folinic acid. *Br. J. Cancer*, **69,** 205 – 211.

FOGH J, WRIGHT WC AND LOVELESS JD. (1977). Absence of HeLa cell contamination in 169 cell lines derived from human tumors. *J. Natl. Cancer Inst.*, **58,** 209 – 214.

GRINDEY GB, ALATI T, LANTZ R, POHLAND R AND SHIH C. (1992). Role of dietary folic acid in blocking the toxicity but not the antitumor activity of Lometrexol (DDATHF) (abstract 216). *Ann. Oncol.*, **3** (suppl.), 113.

HAMILTON TC, YOUNG RC, McKOY WM, GROTZINGER KR, GREEN JA, CHU EW, WHANG-PENG J, ROGAN AM, GREEN WR AND OZOLS RF. (1983). Characterization of a human ovarian carcinoma cell line (NIH:OVCAR-3) with androgen and estrogen receptors. *Cancer Res.*, **43,** 5379 – 5389.

HUMPHREYS A, BAILEY N, LAOHAVINIJ S, SIMMONS D, ROBSON L AND CALVERT A. (1995). Amelioration of toxicity from Lometrexol (DDATHF) using oral folic acid – A phase I study update (abstract P187). *Br. J. Cancer*, **71,** 71.

JANSEN G, WESTERHOF GR, KATHMANN I, RIJKSEN G AND SCHORNAGEL JH. (1991). Growth-inhibitory effects of 5,10-dideazatetrahydrofolic acid on variant murine L1210 and human CCRF-CEM leukemia cells with different membrane-transport characteristics for (anti)folate compounds. *Cancer Chemother. Pharmacol.*, **28,** 115 – 117.

KAMEN BA, JOHNSON CA, WANG MT AND ANDERSON RGW. (1989). Regulation of the cytoplasmic accumulation of 5-methyltetrahydrofolate in MA104 cells is independent of folate receptor regulation. *J. Clin. Invest.*, **84,** 1379 – 1386.

MIOTTI S, CANEVARI S, MENARD S, MEZZANZANICA D, PORRO G, PUPA SM, REGAZZONI M, TAGLIABUE E AND COLNAGHI MI. (1987). Characterization of human ovarian carcinoma-associated antigens defined by novel monoclonal antibodies with tumor-restricted specificity. *Int. J. Cancer*, **39,** 297 – 303.

MIOTTI S, ALBERTI S, FACHERIS P, MANTOVANI L, FORNARO M, STELLA M, MENARD S, CANEVARI S AND COLNAGHI MI. (1992). Membrane association and shedding of the GPI-anchored Ca-MOv18 antigen in human ovary carcinoma cells. *Int. J. Cancer*, **51,** 499 – 505.

MORAN RG, TAYLOR EC AND BEARDSLEY GP. (1985). 5,10-dideaza-5,6,7,8-tetrahydrofolic acid (DATHF), a potent antifolate inhibitory to *de novo* purine synthesis (abstract 910). *Proc. Am. Assoc. Cancer Res.*, **26,** 231.

MORAN RG, BALDWIN SW, TAYLOR EC AND SHIH C. (1989). The 6S- and 6R-diastereomers of 5,10-dideaza-5,6,7,8-tetrahydrofolate are equiactive inhibitors of *de novo* purine synthesis. *J. Biol. Chem.*, **264,** 21047 – 21051.

MUGGIA F, MARTIN T, RAY M, LEICHMAN CG, GRUNBERG S, GILL I, MORAN R, DYKE R AND GRINDEY G. (1990). Phase I study of weekly 5,10-dideazatetrahydrofolate (LY 264618, DDATHF-B) (abstract 285). *Proc. Soc. Clin. Oncol.*, **9,** 74.

PIZZORNO G, SOKOLOSKI JA, CASHMORE AR, MOROSON BA, CROSS AD AND BEARDSLEY GP. (1991). Intracellular metabolism of 5,10-dideaza-5,6,7,8-tetrahydrofolic acid in human leukemia cell lines. *Mol. Pharmacol.*, **39,** 85 – 89.

PIZZORNO G, CASHMORE AR, MOROSON BA, CROSS AD, SMITH AK, MARLING-CASON M, KAMEN BA AND BEARDSLEY GP. (1993). 5,10-dideazatetrahydrofolic acid (DDATHF) transport in CCRF-CEM and MA104 cell lines. *J. Biol. Chem.*, **268,** 1017 – 1023.

PIZZORNO G, MOROSON BA, CASHMORE AR, RUSSELLO O, MAYER JR, GALIVAN J, BUNNI MA, PRIEST DG AND BEARDSLEY GP. (1995). Multifactorial resistance to 5,10-dideazatetrahydrofolic acid in cell lines derived from human lymphoblastic leukemia CCRF-CEM. *Cancer Res.*, **55,** 566 – 573.

RAY MS, MUGGIA FM, LEICHMAN CG, GRUNBER SM, NELSON RL, DYKE RW AND MORAN RG. (1993). Phase I study of (6R)-5,10-dideazatetrahydrofolate: a folate antimetabolite inhibitory to de novo purine synthesis. *J. Natl Cancer Inst.*, **85,** 1154 – 1159.

ROSS JF, CHAUDHURI PK AND RATNAM M. (1994). Differential regulation of folate receptor isoforms in normal and malignant tissues *in vivo* and in established cell lines. Physiologic and clinical implications. *Cancer*, **73,** 2432 – 2443.

SADASIVAN E, CEDENO MM AND ROTHENBERG SP. (1994). Characterization of the gene encoding a folate-binding protein expressed in human placenta. *J. Biol. Chem.*, **269,** 4725 – 4735.

SESSA C, GUMBRELL L, HATTY S, KERN H AND CAVALLI F. (1990). Phase I study of 5,10-dideazatetrahydrofolic acid (Ly264618; DDATHF) given daily for 3 consecutive days. *Ann. Oncol.*, **1** (suppl.), P5:20.

SHIH C, GRINDEY GB, HOUGHTON PJ AND HOUGHTON JA. (1988). *In vivo* antitumor activity of 5,10-dideazatetrahydrofolic acid (DDATHF) and its diastereomeric isomers (abstract 1125). *Proc. Am. Assoc. Cancer Res.*, **29,** 283.

TAYLOR EC, HAMBY JM, SHIH C, GRINDEY GB, RINZEL SM, BEARDSLEY GP AND MORAN RG. (1989). Synthesis and antitumor activity of 5-deaza-5,6,7,8-tetrahydrofolic acid and its $N^{10}$-substituted analogues. *J. Med. Chem.*, **32,** 1517 – 1522.

WESTERHOF GR, JANSEN G, VAN EMMERIK N, KATHMANN I, RIJKSEN G, JACKMAN AL AND SCHORNAGEL JH. (1991). Membrane transport of natural folates and antifolate compounds in murine L1210 leukemia cells: role of carrier- and receptor-mediated. *Cancer Res.*, **51,** 5507 – 5513.

YOUNG C, CURRIE V, BALTZER L, TROCHANOWSKI B, ETON O, DYKE R AND BOWSHER R. (1990). Phase I and clinical pharmacologic study of LY264618, 5,10-dideazatetrahydrofolate (abstract 1053). *Proc. Am. Assoc. Cancer Res.*, **31,** 177.